# Construction of Yeast One-Hybrid Library of *Alternaria oxytropis* and Screening of Transcription Factors Regulating swnK Gene Expression

**DOI:** 10.3390/jof9080822

**Published:** 2023-08-03

**Authors:** Jiaqi Xue, Haodong Zhang, Qingmei Zhao, Shengwei Cui, Kun Yu, Ruohan Sun, Yongtao Yu

**Affiliations:** 1 School of Animal Science and Technology, Ningxia University, Yinchuan 750021, China; xjq19970307@163.com (J.X.);; 2 College of Biological Science and Engineering, North Minzu University, Yinchuan 750021, China; 3 Ningxia Key Laboratory of Ruminant Molecular and Cellular Breeding, School of Animal Science and Technology, Ningxia University, Yinchuan 750021, China

**Keywords:** swainsonine, swnK gene, yeast one-hybrid, transcriptional regulator molecules

## Abstract

The indolizidine alkaloid-swainsonine (SW) is the main toxic component of locoweeds and the main cause of locoweed poisoning in grazing animals. The endophytic fungi, *Alternaria* Section *Undifilum* spp., are responsible for the biosynthesis of SW in locoweeds. The swnK gene is a multifunctional complex enzyme encoding gene in fungal SW biosynthesis, and its encoding product plays a key role in the multistep catalytic synthesis of SW by fungi using pipecolic acid as a precursor. However, the transcriptional regulation mechanism of the swnK gene is still unclear. To identify the transcriptional regulators involved in the swnK gene in endophytic fungi of locoweeds, we first analyzed the upstream non-coding region of the swnK gene in the *A. oxytropis* UA003 strain and predicted its high transcriptional activity region combined with dual-luciferase reporter assay. Then, a yeast one-hybrid library of *A. oxytropis* UA003 strain was constructed, and the transcriptional regulatory factors that may bind to the high-transcriptional activity region of the upstream non-coding region of the swnK gene were screened by this system. The results showed that the high transcriptional activity region was located at −656 bp and −392 bp of the upstream regulatory region of the swnK gene. A total of nine candidate transcriptional regulator molecules, including a C2H2 type transcription factor, seven annotated proteins, and an unannotated protein, were screened out through the Y1H system, which were bound to the upstream high transcriptional activity region of the swnK gene. This study provides new insight into the transcriptional regulation of the swnK gene and lays the foundation for further exploration of the regulatory mechanisms of SW biosynthesis in fungal endophytic locoweeds.

## 1. Introduction

Locoweed is a general term for poisonous plants belonging to the genus *Oxytropis* and *Astragalus* of the family Leguminosae, which are widely distributed in arid and semi-arid regions of China, the United States, and Canada [1,2]. Chronic toxic disease characterized by nervous system dysfunction occurs in grazing animals after excessive consumption of locoweeds, resulting in clinical symptoms such as ataxia, paralysis, infertility, abortion, and eventually death [3,4,5]. The indolizidine alkaloid swainsonine (SW) is the main toxic component of locoweeds and the main cause of livestock poisoning [6,7]. Studies have shown that the endophytic fungi of locoweeds, *A. oxytropis*, are responsible for the production of swainsonine in locoweed [8,9,10,11].

It has been shown that the synthetic pathway of SW in the endophytic fungi of locoweed may be similar to those of the insect pathogen *M. robertsii* and the clover pathogen *S. leguminicola*. Further studies have shown that there are type I polyketide synthase (T1-PKS) gene clusters with high orthologs in the swainsonine-producing fungi, including *A. oxytropis*, *S. leguminicola*, *M. robertsii*, *Chaetothyriaceae* spp., *Trichophyton* spp., and *Arthroderma* spp., etc [8,12,13,14]. The gene cluster has been confirmed to be essential for the biosynthesis of swainsonine in *M. robertsii*. Therefore, the gene clusters in the fungi producing swainsonine are designated swainsonine-biosynthesis gene clusters (SWN) [14]. SWN mainly consist of swnA, swnH1, swnH2, swnK, swnN, swnR, and swnT (swnA and swnT are absent in *A. oxytropis*), among which swnK is the most important gene, consisting of five domains: A, T, KS, AT, and SDR. The swnK genes of *A. oxytropis*, *S. leguminicola,* and *M. robertsii* share more than 70% identity with each other and are hypothesized to encode the multifunctional enzymes that are responsible for the catalytic reactions of the first steps of the swainsonine synthesis in fungi using pipecolic acid as a precursor [15].

*Alternaria* Section *Undifilum* spp. contains non-pathogenic *Alternaria* endophytes *A. oxytropis*, *A. fulva*, and *A. cinereum* from locoweeds and plant pathogenic fungi *A. bornmuelleri* and *A. gansuense* [10,16]. Although the microscopic morphology of these strains is similar, and the swnK genes sequence is highly consistent, there were still significant differences in SW synthesis among different strains [6,17,18,19]. For example, plant pathogenic fungi such as *A. bornmuelleri* and *A. gansuense* produce only minimal SW [20,21]. However, there have been no reports on the regulation of swnK gene expression, and the transcriptional regulation mechanism of swnK gene is still unclear. 

In the regulation of gene expression in fungi, transcriptional regulatory molecules such as transcription factors regulate the manner and intensity of gene expression by interacting with the regulatory sequences in the non-coding regions of target genes. The yeast one-hybrid (Y1H) approach, which was developed based on the basic principle of protein-DNA interaction, has been widely used to screen transcription regulatory molecules involved in the regulation of gene expression [22,23,24]. To identify the transcriptional regulators involved in the swnK gene in endophytic fungi of locoweeds, we first analyzed the upstream non-coding region of the swnK gene, and, combined with the results of the dual luciferase reporter assay, we predicted that the high transcriptional activity region is located between −656 bp and −392 bp upstream of the swnK gene. Then, a yeast one-hybrid library of *A. oxytropis* UA003 strain was constructed, and the transcriptional regulatory factors that may bind to the high-transcriptional activity region of the upstream non-coding region of the swnk gene were screened by this system. The structure and function of the selected transcription regulatory molecules were analyzed. The results will provide new clues for the transcriptional regulation mechanism of the swnK gene and also lay a foundation for further exploration of the regulatory mechanism of SW biosynthesis in the endophytic fungi of locoweeds.

## 2. Materials and Methods

### 2.1. Fungal Culture

*A. oxytropis* UA003 was isolated from *Astragalus variabilis* and identified by Xiaonan Bai et al. [25]. The strains were first resuscitated, and the preserved UA003 strain samples were taken out of the freezer (−80 °C), thawed on ice, and inoculated on potato dextrose agar (PDA) plates at 25 °C for 15 days. Then the mycelia were picked and re-inoculated in PDA plates at 25 °C for 30 days.

### 2.2. Prediction and Cloning of the 5′ Non-Coding Region of the swnK Gene

PlantCare was used to analyze the cis-acting elements of the upstream regulatory region of the swnK gene (http://bioinformatics.psb.ugent.be/webtools/plantcare/html/, accessed on 23 November 2022) [26]. The promoter core region of the swnK gene was predicted by Neural Network Promoter Prediction (https://www.fruitfly.org/seq_tools/promoter.html, accessed on 23 November 2022) [27]. Laboratory of Molecular Medicine (https://www.urogene.org/cgi-bin/methprimer/methprimer.cgi, accessed on 23 November 2022) was performed to make predictions for CpG islands [28].

The genome DNA of *A. oxytropis* UA003 was obtained using an E.Z.N.A.^®^ Fungal DNA Kit (Omega Bio-Tek, GA, USA). The gene-specific primers (swnK-PF1/PR, Table 1) were designed to amplify a fragment of approximately 1.2 kb in size in the 5′ non-coding region of the swnK gene (NCBI accession: KY365741.1). The PCR reaction mixture consisted of 25 μL Premix Taq (TaKaRa, Dalian, China), each 1 μL upstream and downstream primers and templates, and ddH2O was added to 25 μL. Thermal cycling consisted of initial denaturation at 94 °C for 5 min, followed by 30 cycles of denaturation at 94 °C for 30 s, annealing at 60 °C for 30 s, and elongation at 72 °C for 30 s. Finally, 72 °C for 10 min. PCR products were purified with E.Z.N.A.^®^ Gel Extraction Kit (Omega) ligated into the pMD19-T vector and subsequently transformed into DH5α competent cells. The positive clones were selected and sent to Sangon Bioengineering Co., Ltd. (Shanghai, China) for sequencing.

### 2.3. Analysis of Transcriptional Activity in the 5′ Upstream Regulatory Region of swnK Gene

To identify regions of high transcriptional activity in the 5′ upstream regulatory region of the swnK gene, five pairs of specific primers with KpnI and BglII restriction sites, swnK-PF1/R (−1152/+69), swnK-PF2/R (−891/+69), swnK-PF3/R (−656/+69), swnK-PF4/R (−392/+69), and swnK-PF5/R (−209/+69), were designed to amplify five unidirectional deletions of 5′ upstream regulatory region of swnK gene (Table 1). The amplified fragment was cloned into the pMD19-T vector and digested using KpnI and BglII restriction enzymes. The digestion products were purified and then cloned into the corresponding restriction sites of the pGL3-Basic vector to generate five luciferase reporter constructs with unidirectional deletions of the 5′ upstream regulatory region of the swnK gene. After sequencing verification, extracted five luciferase reporter constructs with the endotoxin-free plasmid extraction kit (TIANGEN, Beijing, China) and stored at −20 °C for subsequent transformation.

The dual luciferase reporter (DLR) system was used to assess the transcriptional activity of the five unidirectional deletions [29]. HEK-293T cells were inoculated in 24-well plates with DMEM complete medium containing 10% fetal bovine serum and antibiotics (100 IU/mL penicillin and 100 µg/mL streptomycin) at 37 °C and 5% CO_2_ in a cell incubator. Luciferase reporter constructs and internal reference plasmid pRL-TK at the ratio of 24:1 were co-transformed into HEK-293T cells with the assistance of Lipofectamine 3000 Reagent (Invitrogen, Carlsbad, CA, USA). At the same time, pGL3-Basic vectors were used as the negative control. Forty-eight hours after transfection, luciferase activity was measured using the Dual-Luciferase Reporter Assay System (Promega, Madison, WI, USA) according to the manufacturer’s instructions. Relative luciferase activity was the ratio of firefly luciferase relative light unit (RLU) to Renilla luciferase RLU. Three parallel replicates were required in all experiments.

All values were shown as mean ± standard deviation, and the analysis of the variance of the experimental data was processed by SPSS20.0 software. LSD-t test was used for comparison between groups. *p* < 0.05 was considered a significant difference, and *p* < 0.01 was considered an extremely significant difference.

### 2.4. RNA Extraction and Yeast One-Hybrid (Y1H) Library Construction

According to the manufacturer’s instructions, the total RNA of the *A. oxytropis* UA003 strain was extracted using Trizol Reagent (Invitrogen), and then mRNA was isolated from the total RNA using Oligotex mRNA Midi Kit (Qiagen, Hilden, Germany). The double-stranded cDNA was synthesized using the CloneMiner II cDNA Library Construction kit (Thermo Fisher Scientific, Wilmington, USA). After ligation with the three-frame attB1 recombinant adaptor, the cDNA was graded and collected.

The Y1H library was constructed using Gateway technology. First, the Y1H primary library was constructed by BP recombination reaction. The cDNA was cloned into the pDONR222 vector and incubated with BP Clonase ^®^ II enzyme mix (Invitrogen) for 16–20 h. The mixture was electroporated into *E. coli* competent cells DH10B for culture. The E.Z.N.A. ^®^ Plasmid Maxi Kit (Omega) was used to extract primary library plasmids from transformed cultures. Then the Y1H secondary library was constructed by LR recombination reaction. The primary library plasmids were cloned into pGADT7-DEST and incubated with LR Clonase ^®^ II enzyme mix (Invitrogen) for 16–20 h. The mixture was electroporated into *E. coli* competent cells DH10B for culture. The positive clones were collected and amplified, and the yeast secondary library was successfully constructed. The secondary library bacterial solution was added with glycerol for preservation, and the plasmid was extracted for subsequent experiments.

The quality of Y1H was mainly identified by library titer, recombination rate, and average length of inserted fragments. The identification method of the titer of the library was as follows: 10 μL of the secondary library bacterial solution SOC medium was diluted 100 times, and then 50 μL of the diluted bacterial solution was spread on LB (containing 100 μg/mL ampicillin) and cultured overnight at 37 °C. The library titer was calculated using the following formula. Twenty-four clones were randomly selected from the solid medium and identified by PCR using primers T7/3’AD (Table 1).

CFU/mL = number of clones on the plate/50 μL × 100 multiple × 10^3^ μL (1)

Library total CFU = CFU/mL × total volume of the library bacterial solution (mL)(2)

### 2.5. Construction of Bait Strain and Self-Activation Identification

The 5′ upstream high transcriptional activity fragment (−656~−392bp) of the swnK gene was amplified by using the *A. oxytropis* UA003 genome as a template and primer hp-F/R (Table 1). The high transcriptional activity region was digested by restriction endonucleases KpnI and XhoI (TaKaRa) and cloned into the corresponding restriction site of the pAbAi vector to construct the bait vector pAbAi-hp. The bait plasmid pAbAi-hp and the positive control plasmid p53-AbAi were digested with restriction endonuclease BstB I (NEB, Ipswich, USA), integrated into the Y1HGold cells by PEG/LiAc method, and transferred to SD/-Ura plate for incubation at 30 °C for 2–3 days [30]. YP-hp-F/R and p53-F/R primers (Table 1) were used to confirm whether the recombinant plasmid was correctly integrated, that is, whether the bait strain was successfully constructed.

Aureobasidin A (AbA) is a cyclic ester peptide antibiotic; a low concentration of AbA can inhibit the growth of yeast Y1HGold. The bait strain contains the AbA resistance gene (AUR1-C). When the prey protein interacts with the bait fragment, GAL4 AD activates the expression of the AUR1-C gene, and the bait yeast obtains AbA resistance. Autoactivation testing of the bait strain is required before screening cDNA libraries to ensure that prey proteins are derived from cDNA libraries rather than endogenous yeast proteins, and should strictly test the minimum AbA concentration of the bait yeast strain. The bait and positive control strains were spread on SD/-Ura (containing different AbA concentrations) plates and incubated at 30 °C for 3–5 days. The lowest concentration of AbA inhibiting the growth of the strain was identified and used for subsequent library screening.

### 2.6. Screening for Candidate Transcriptional Regulatory Molecules of swnK Gene

The bait strain was prepared into competent cells by the LiAc method, and 10 μg of yeast library plasmid was transformed into 600 μL of competent cells of the bait strain. The 150 μL transformed bacterial liquid was spread on the SD/-Leu agar screening plate containing AbA and cultured at 30 °C for 3–5 d. When the interaction clone grew to 1–2 mm, they were transferred to the SD/-Leu agar screening plate with the same AbA concentration for culture. The interaction clones with a diameter greater than 2 mm were selected for PCR identification using the primer T7/3’AD (Table 1). The amplified products were purified for nucleic acid sequencing. The sequencing results were subjected to bioinformatics analysis by NCBI blast X to obtain candidate transcriptional regulatory molecules of the swnK gene.

### 2.7. Confirmation of Positive Interaction

The positive clone plasmid was extracted and transformed into the bait strain for one-to-one verification to confirm the candidate transcriptional regulators interacting with the upstream high transcriptional activity region of the swnK gene. The yeast plasmid DNA of the interaction clone was extracted using the UNlQ-10 column yeast plasmid DNA extraction kit (Sangon Bioengineering Co., Ltd., Shanghai, China), then transformed into DH5α competent cells for amplification culture. TIANprep Mini Plasmid Kit (TIANGEN) was used to extract the amplified plasmid, which is the prey plasmid. The prey plasmid and pGADT7 empty vector (negative control) were transformed into Y1H [pAbAi-hp] bait strain competent cells and spread on SD/-Leu and SD/-Leu/AbA plates, respectively, for 3–5 d. According to the growth results, the interaction was confirmed one by one. If it grew normally on SD/-Leu, it indicated that the transformation system was functional. If it grew on SD/-Leu/AbA plate, it indicated that the candidate factor could interact with the high transcriptional activity region of the swnK gene.

## 3. Results

### 3.1. Identification of Highly Transcriptively Active Regions in the 5′ Non-Coding Region of the swnK Gene

**Figure 1:**
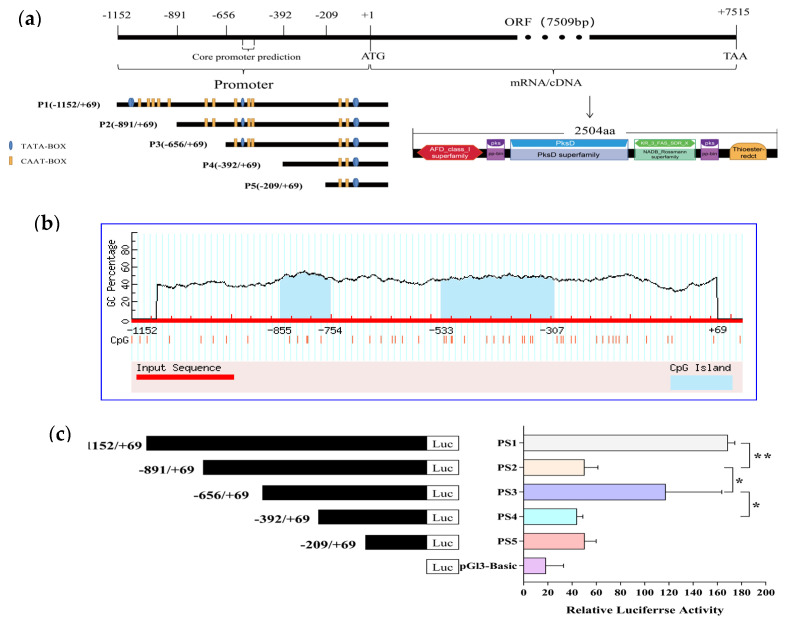
Structural characteristics of swnK gene and identification of its core promoter in *A. oxytropis*. (**a**). The detailed genome, mRNA, protein conserved domains, cis-acting elements TATA-BOX, CAAT-BOX positions and predicted promoter positions of swnK gene. (**b**). The CpG island prediction map of swnK gene promoter region shows that the red X axis represents the swnK gene promoter region, the Y axis represents the GC percentage, and the blue pre-region is the CpG island. (**c**). Activity of swnK promoter in HEK293T cells detected by DLR; * *p* < 0.05 indicated significant difference; ** *p* < 0.01 means extremely significant difference.

The 5′ non-coding region of the swnK gene of the *A. oxytropis* UA003 strain was amplified by PCR (about 1.2 kb). The sequence was compared with the published sequence of *A. oxytropis* (KY365741.1) by the NCBI-BLAST program, and the consistency was 99.9%. The prediction of the 5’ non-coding region of the swnK gene showed that the sequence contained some core promoter elements such as 5 TATA-BOXes and 12 CAAT- BOXes (Figure 1a). There was a core promoter fragment between −577 bp and −527 bp, namely a high transcriptional activity region (Figure 1a). In addition, two CpG islands were predicted, located from −855 bp to −754 bp and from −533 bp to −307 bp (Figure 1b).

The results of the DLR assay (relative luciferase activity) represent relative transcriptional activity (Figure 1c). Transcriptional activity analysis indicated that the five luciferase reporter constructs with unidirectional deletions of the 5′ upstream regulatory region showed significantly higher luciferase activity than the negative control. The recombinant plasmid pGL3-basic-P1 containing the full-length 5′ upstream regulatory region showed the highest relative transcriptional activity. Compared with pGL3-basic-P1, the relative luciferase activity of pGL3-basic-P2 was reduced by 70.4% (*p* < 0.01). However, pGL3-basic-P3 had significantly higher relative luciferase activity than pGL3-basic-P2 (*p* < 0.05) and pGL3-basic-P4 (*p* < 0.05). The results indicated that deletion of the sequence from −891 to −656 increased basal transcriptional activity approximately twofold (*p* < 0.01), suggesting that this region may contain negative regulatory elements. The significantly increased transcriptional activity of pGL3-basic-P3 suggested the presence of positive regulatory elements in the region between −656 and −392 that may be critical for the transcription of the swnK gene. Therefore, the region between −656 and −392 of the 5′ upstream regulatory region was identified as high transcriptional activity region, consistent with the prediction.

### 3.2. Construction of Y1H Library of A. Oxytropis UA003

**Figure 2:**
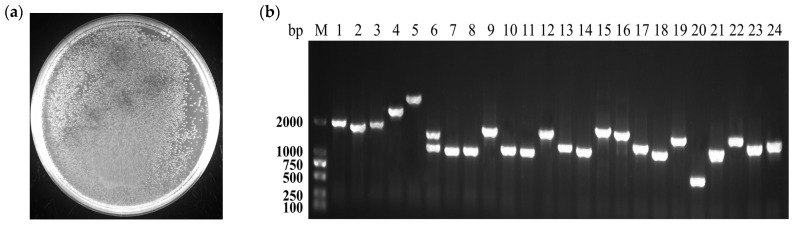
Quality inspection of UA003 yeast one-hybrid library. (**a**). The bacterial solution of the secondary library was diluted 100 times and spread with plate count. (**b**). 24 clones were randomly selected to identify the recombination rate and the average length of the inserted fragments by colony PCR.

Y1H library of *A. oxytropis* UA003 strain was successfully constructed using Gateway technology in this study. About 2000 clones containing secondary library plasmids were grown in petri dishes with a titer of 1.6 × 10^7^ CFU/mL (Figure 2a). Twenty-four clones were randomly selected for colony PCR identification, and the results showed that the length of inserted fragments was mainly 750 bp~2000 bp, and the recombination rate reached 100% (Figure 2b).

### 3.3. Construction of Bait Strain and Self-Activation Identification Results

**Figure 3:**
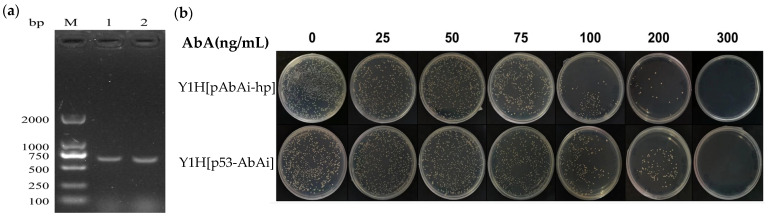
The bait strain and the verification of its self-activation. (**a**). Colony PCR identification of Y1HGold [pAbAi-hp] bait strain, M lane: 2-kb DNA Marker; 1-2 lane: Y1HGold [pAbAi-hp] bait strain colony PCR, YP-hp-F/R primers were used for amplification, and the band size was about 638 bp. (**b**). Self-activation verification of bait strain, Y1HGold [pAbAi-hp] was the bait strain and Y1HGold [p53-AbAi] was the positive control strain.

The bait strains were identified by colony PCR using YP-hp-F/R primers (primers on the vectors at both ends of the target fragment). As shown in Figure 3a, it yielded a fragment of about 638 bp, as expected. As confirmed by sequencing, the bait strain was successfully constructed. The identification results of autoactivation are shown in Figure 3b. The bait strain Y1H [pAbAi-hp] did not grow on the medium with an AbA concentration of 300 ng/mL, indicating that the strain did not autoactivate, and the AbA concentration used in the subsequent library screening test was established to be 300 ng/mL.

### 3.4. Screen Potential Transcriptional Regulators in the 5′ Highly Transcriptional Active Region of the swnK Gene Using Y1H

Y1H library was screened by bait strain with 5′ upstream high transcriptional activity fragment (−656~−392bp) of the swnK gene. Although Y1H is a well-established and common method, it is associated with a high rate of false positives and sequence reproducibility, which requires two rounds of screening. After the initial screening, 108 positive clones were obtained. These 108 positive clones were transferred to the same screening medium for secondary screening, and 31 positive clones grew again. Subsequently, 12 sequences were finally identified from the screen by sequencing and removing repetitive sequences. These sequences were compared by BLAST X, and 11 sequences were annotated to different transcriptional regulators (Table 2), one of which was a C2H2 transcription factor protein. In addition, no information annotation was obtained for h12.

### 3.5. Verification of the Interaction between 5’ Upstream High Transcriptional Activity Region of the swnK Gene and Candidate Transcriptional Regulatory Molecules

**Figure 4:**
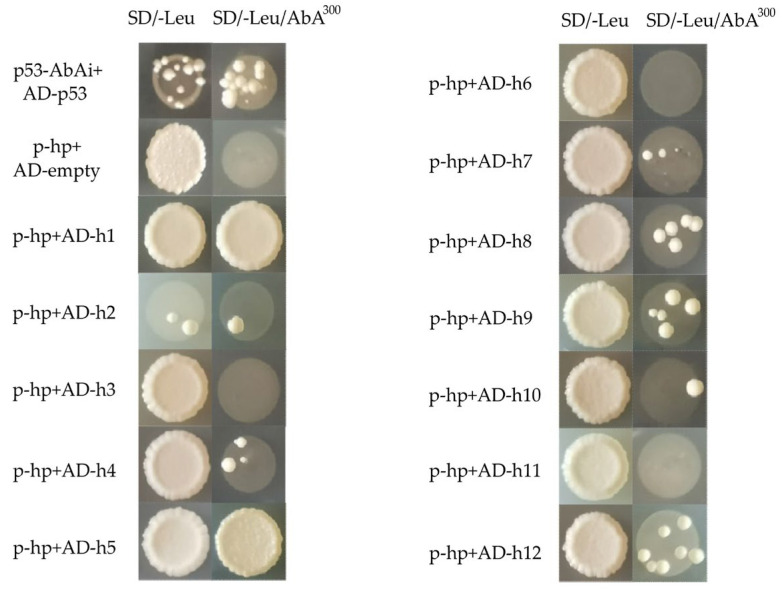
The interaction between candidate factors and 5′ highly transcriptionally active region upstream of the swnK gene was verified by Y1H reverse, The empty vector of AD was transferred into Y1HGold [pAbAi-hp] as a negative control, and AD-p53 was transferred into Y1HGold [p53-AbAi] as a positive control, AD-h1~AD-h12 were library plasmids extracted from positive clones obtained by secondary screening and transformed into bait strain Y1HGold [pAbAi-hp] for one-to-one interaction verification.

To further confirm the interaction between the candidate factors and the 5′ high transcriptional activity region upstream of the swnK gene, the plasmids containing the 12 candidate factors were transformed into Y1H [pAbAi-hp] competent cells for Y1H validation. The results showed (Figure 4) that all strains could grow on SD/-Leu plates, indicating no problem with the Y1H transformation system used. Strains labeled h1, h2, h4, h5, h7, h8, h9, h10, and h12 (information annotation not obtained) could grow again on SD/-Leu/AbA^300^ plates. This indicates that these strains correspond to transcriptional regulators (C2H2 transcription factor protein, uncharacterized protein, RING-14 protein, histone H3 protein, tuberous sclerosis 1 protein, eukaryotic translation initiation factor 2 subunit alpha, high osmolarity signaling protein sho1, bli-3 protein) could interact with the highly transcriptional active region in the 5′ high transcriptional activity region upstream of swnK gene. In addition, h1 and h5 strains grew more vigorously under the same culture conditions and time. It is speculated that the corresponding C2H2 transcription factors and histone H3 may interact more strongly with the upstream non-coding region of the swnK gene.

### 3.6. Bioinformatics Analysis of the Selected C2H2 Transcription Factors

**Figure 5:**
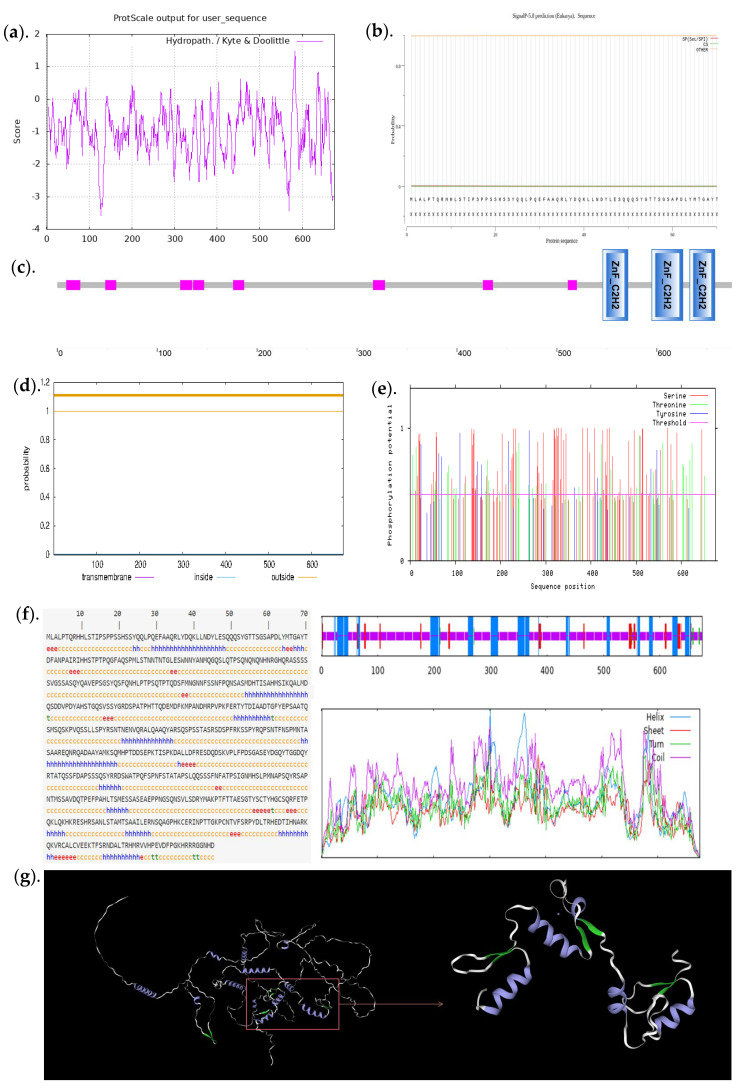
Bioinformatics analysis of the selected C2H2 transcription factors. (**a**). Prediction of hydrophilicity. (**b**). Signal peptide prediction. (**c**). Conserved domain prediction, the pink box is the low complexity area. (**d**). Prediction of transmembrane structure. (**e**). Phosphorylation site prediction. (**f**). Protein secondary structure prediction. (**g**). Prediction of protein tertiary structure, the green region is β-sheet structure, and the blue region is α-helix structure.

The C2H2 transcription factors that interacted with the swnK gene screened by Y1H were analyzed by bioinformatics. The CDS sequence of this C2H2-type transcription factor is 2028bp in length and encodes 675 amino acids. Expasy Pro Param (https://web.expasy.org/protparam/, accessed on 29 March 2023) prediction results showed that the molecular weight was 74488.91, the theoretical isoelectric point was 6.68, the instability coefficient was 67.36, the fat index was 39.16, and the average hydrophilic coefficient was −0.989. The predicted results of ProScale (https://web.expasy.org/protscale/, accessed on 8 April 2023) are shown in Figure 5a. Amino acid 127 is the most hydrophilic (score −3.578), and amino acid 582 is the most hydrophobic (score 1.489). Since the hydrophilic region is larger than the hydrophobic region, the protein is presumed to be hydrophilic. The SMART database (http://smart.embl-heidelberg.de/, accessed on 8 April 2023) predicted that the gene contained three C2H2 conserved domains (Figure 5c). Signal P 5.0 (https://services.healthtech.dtu.dk/services/SignalP-5.0/, accessed on 8 April 2023) to predict the C2H2 transcription factor protein no Signal peptide (Figure 5b). TMHMM2.0 (http://www.cbs.dtu.dk/services/TMHMM/, accessed on 8 April 2023) to predict the C2H2 transcription factor protein has no transmembrane structure (Figure 5d). The prediction results of Net Phos 3.1 Server (http://www.cbs.dtu.dk/services/Netphos, accessed on 8 April 2023) showed that the protein contained multiple phosphorylation sites, including 13 tyrosine (Tyr) phosphorylation sites, 37 threonine (Thr) phosphorylation sites, and 75 serine (Ser) phosphorylation sites (Figure 5e). These sites may be phosphorylated to regulate the activity and function of the transcription factor protein. The results of protein secondary structure prediction (SOPMA: http://npsa-pbil.ibcp.fr/cgi-bin/npsa_automat.pl?page=npsa_sopma.html, accessed on 29 April 2023) showed that alpha helix (Hh) accounted for 19.70%, extended strand (Ee) accounted for 5.78%, beta-turn (Tt) accounted for 1.04%, and that random coil (Cc) accounted for 73.48% in the C2H2 transcription factors (Figure 5f). SWISS-MODEL (https://swissmodel.expasy.org/interactive/, accessed on 1 May 2023) was used to predict C2H2 transcription factor protein tertiary structure (Figure 5g). The Seq Identity of the constructed tertiary structure was 85.35%, which was mainly composed of α-helix and random coil structure, while the extended chain and β-turn were less, which was consistent with the prediction of the secondary structure of the protein encoded by the C2H2 transcription factor gene. From the correct magnification of Figure 5g, it can be seen that the C2H2 type transcription factor protein contains three typical C2H2 zinc finger type structures located between amino acid sequence 542 and 658 regions; this is consistent with the predicted protein domain. Each C2H2 structure has a pair of anti-parallel β-sheets and an α-helix, which conforms to the standard C2H2 spatial structure. C2H2 chelates Zn^2+^ by two conserved His at the C terminus of the α helix and two conserved Cys at the β-fold terminus to form a tetrahedral structure, each of which contains a small Zn^2+^ containing structure.

## 4. Discussion

SW-producing fungi, *Alternaria* Section *Undifilum* spp., *S. leguminicola*, *M. robertsii*, and *Chaetothyriaceae* spp., share more than 70% sequence consistency with the swnK gene. The gene has been confirmed to be required for the biosynthesis of SW in *M. robertsii* [14,31]. Recent research has shown that the expression of the swnK gene in *A. oxytropis* was closely related to the synthesis of SW [32]. Although different species of *Alternaria* Section *Undifilum* share highly homologous swnK genes, there are significant differences in their ability to synthesize SW. For example, the swnK gene sequences of *A. bornmuelleri* and *A. gansuense* are highly similar to those of *A. oxytropis*, but these two species only produce very low amounts of SW. At present, the regulatory mechanism of SW biosynthesis in fungi remains unclear. Understanding the transcriptional regulation of the swnK gene is helpful in elucidating the regulatory mechanism of SW biosynthesis in fungi.

At the transcriptional level, gene expression in eukaryotes is mainly regulated by cis-acting elements, trans-acting factors, and RNA polymerases [33,34]. Cis-acting elements mainly include non-coding regulatory sequences such as promoters, enhancers, and repressors located upstream or downstream of gene coding regions, while trans-acting factors are some transcriptional regulatory factors that can interact with cis-acting elements [35,36,37]. Generally, high transcriptional activity regulatory sequences, including the core promoter in the non-coding region of the gene, interact with transcriptional regulatory factors to regulate the expression patterns and levels of coding genes at different times and spaces at the transcriptional level and are frequently used to screen transcriptional regulatory factors involved in the regulation of target gene expression [38,39]. In this study, the 5′ non-coding region of the swnK gene of *A. oxytropis* UA003 was conducted for bioinformatics analysis and transcriptional activity test. The results showed that the −577 bp~−527 bp in the 5′ non-coding region of the swnK gene had the characteristics of core promoter, and −656 bp to −392 bp region in the 5′ non-coding region had high transcriptional activity and multiple TATA-boxes and CAAT-boxes. TATA-box and CAAT-box are important core promoter elements that can regulate the accurate transcription and transcription efficiency of genes [40]. In addition, there was a CpG island between −533 and −307 bp in the 5′ non-coding region of the swnK gene. The CpG island and its methylation status can regulate the activity of certain promoters [41].

Transcription factors and other trans-acting factors participate in the transcriptional regulation of gene expression through interaction with non-coding regions of target genes and have important effects on fungal growth, development, and metabolism [42]. In this study, the Y1H system and the highly transcriptionally active region of the swnK gene were used to screen the regulators that may be involved in the transcriptional regulation of the swnK gene in the *A. oxytropis* UA003 strain. The results showed that the C2H2 transcription factor, uncharacterized protein, RING-14 protein, histone H3 protein, tuberous sclerosis 1 protein, eukaryotic translation initiation factor 2 subunit alpha, high osmolarity signaling protein sho1, and bli-3 protein may be involved in the biosynthesis of SW by regulating swnK gene transcription. Among the reported transcriptional regulators, C2H2 has been proven to be a type of common transcription factor in eukaryotes and is involved in the regulation of fungal growth, sporulation, and secondary metabolite synthesis [43,44]. Tao Liu et al. reported that C2H2 zinc finger transcription factor VdCf2a is involved in the regulation of mycelial growth, melanin synthesis, and virulence of *Vibrio dahliae* [45]. The C2H2 transcription factor deletion strain of *Aspergillus flavus* show slower growth and development and reduced conidial production and aflatoxin synthesis [46]. In this study, a C2H2 transcription factor interacting with the highly transcriptionally active region of the 5′ non-coding region of the swnK gene has been frequently observed. The coding sequence of the C2H2 transcription factor in *A. oxytropis* UA003 strains consisted of 2028 nucleotides encoding 675 amino acids. The results of bioinformatics analysis showed that the C2H2 transcription factor protein did not contain signal peptides and transmembrane domains, suggesting that the protein could not have a transmembrane transport function. The analysis results of Cell-Ploc (http://www.csbio.sjtu.edu.cn/bioinf/Cell-PLoc-2/, accessed on 23 April 2023) and WoLF PSORT (https://www.genscript.com/wolf-psort.html, accessed on 23 April 2023) indicated that the protein could be located in the nucleus and may belong to the intramembrane protein. Further prediction showed that the protein contained multiple phosphorylation sites. Protein phosphorylation, as a common post-translational modification, is considered to play an important role in regulating protein function and many physiological activities of cells [47,48]. The tertiary structure prediction analysis of the protein showed that there were three C2H2 conserved domains in the 542~658 amino acid sequence region. C2H2 conserved domain is considered an important guarantee for the function of the C2H2 transcription factor. These results laid a foundation for further elucidating the function of the transcription factor and its role in the transcriptional regulation of the swnK gene.

In this study, other transcriptional regulators were screened. Histone H3 is one of the important components of chromatin, and its amino acid sequence is very conservative. Histone acetylation usually occurs at the N-terminal lysine residues of H3 and H4. This modification neutralizes the positive charge of histones and weakens their interaction with DNA, making it easier for transcription factors to bind to DNA [49,50]. This may be one of the reasons why h5 strains showed stronger interactions in the one-to-one validation assay. Histone acetylation is often associated with transcriptional activation, and it has been demonstrated in fungi that histone H3 can regulate the expression of secondary metabolite synthesis gene clusters by modifying chromatin [51,52]. Eukaryotic translation initiation factor 2 subunit alpha (eIF2α) is one of the necessary proteins for protein translation in eukaryotic cells, which can ensure that mRNA is correctly encoded on the ribosome to form complexes. Studies on eIF2α in fungi have focused on yeast, where serine at position 51 on eIF2α is phosphorylated by the protein kinase GCN2 when cells are short of amino acids, which in turn stimulates the translation of GCN4 mRNA. GCN4 encodes a transcriptional activator of amino acid biosynthesis genes whose expression is under general amino acid control. When amino acids are abundant, translation of GCN4 mRNA is inhibited by short upstream open reading frames (uORFs) in its preconductor [53,54]. Hypertonic signaling protein sho1 is an important sensing membrane protein, which was first discovered and identified in the isolation of hypertonic stress-sensitive mutants. With the deepening of research, sho1 is also found to be an important sensing protein upstream of the MAPK signaling pathway, sensing and activating the external signal [55,56]. Sho1 plays an important role in many aspects of fungi, such as hyphal growth, osmotic regulation, maintenance of cell wall integrity, oxidative stress, temperature regulation, and formation of secondary metabolites [57,58,59]. BLI-3 protein can respond to light induction and then play a role through the mitogen-activated protein (MAP) kinase pathway to achieve the effect on the growth of fungi and the synthesis of secondary metabolites [60,61]. The functions of RING-14 protein and tuberous sclerosis 1 protein in fungi are unknown. The function and role of uncharacterized proteins also need to be further explored.

In the current study, we finally screened out nine transcriptional regulatory molecules in the high transcriptional activity region upstream of the swnK gene. These transcriptional regulatory factors, especially C2H2 transcription factors, may play an important role in the transcriptional regulation of the swnK gene. At present, we have only preliminarily analyzed these transcriptional regulatory factors, and their exact role in the regulation of swnK gene expression remains unclear. Further studies on the molecular structural characteristics and functions of these transcriptional regulatory factors are needed, which will lay a foundation for further revealing the molecular regulatory mechanism of SW synthesis in endophytic fungi of locoweed.

## 5. Conclusions

In this study, the transcriptional activity of the upstream regulatory region of swnK gene in *A. oxytropis* UA003 was identified by dual luciferase reporter system. A high quality yeast one-hybrid library of *A. oxytropis* UA003 was constructed using Gateway technique. Nine potential transcriptional regulatory factors involved in the regulation of swnK gene expression and specific expression candidate proteins were selected from yeast single hybridization library by using bait strains constructed based the upstream of swnK gene with high transcriptional activity sequences. This study is the first to report transcriptional regulatory molecules that may be in-volved in the regulation of swnK gene expression, provides new insight into the transcriptional regulation of the swnK gene and lays the foundation for further exploration of the regulatory mechanisms of SW biosynthesis in fungal endophyte in locoweeds.

## Figures and Tables

**Table 1:** Primers used in this study.

Reaction	Name	Primer Sequence(5′ to 3′)
Promoter cloning	swnK-PF1	CGGGGTACCTCGATAGCAGGTAGCAAGCG
	swnK-PF2	CGGGGTACCTGCCCCTTATCTCATTTCTACTGG
	swnK-PF3	CGGGGTACCCCACGCCTCTTGAGCTATCC
	swnK-PF4	CGGGGTACCTGGCACATGCCTGTCCTATG
	swnK-PF5	CGGGGTACCCGAAAGTTGCAGGGCGATTC
	swnK-R	GGAAGATCTGTCGTGGTCTGAGATGGCTT
Primary library identification	pDONR222-F	GTAAAACGACGGCCAG
	pDONR222-R	CAGGAAACAGCTATGAC
Secondary library identification	T7	TAATACGACTCACTATAGGGC
	3′AD	AGATGGTGCACGATGCACAG
For amplification of hp bait sequence	hp-F	CGGGGTACCCCCCGGTTCCACCAAGTTTT
	hp-R	CCGCTCGAGTCGTGCTCATAGGACAGGCA
Identification of bait vector and strain	YP-hp-F	GAGAGCAACCATCAAGCACC
	YP-hp-R	TGTTAGGATGGGCAAGGCAT
Identification of p53-AbAi positive control vector and strain	p53-F	GTTCCTTATATGTAGCTTTCGACAT
	p53-R	GCGTGTCTATAGAAGTATAG

**Table 2:** Annotation of the positive insert sequences in yeast one-hybrid screening.

Number	NCBI Number	Annotation Information	Species	Number of Clones
h1	OWY48836.1	C2H2 transcription factor protein	*Alternaria alternata*	14
h2	XP_043173239.1	uncharacterized protein	*Alternaria atra*	2
h3	KAH6861725.1	mitochondrial F1-F0 ATP synthase subunit F of fungi-domain-containing protein	*Alternaria alternata*	2
h4	KAH6859764.1	RING-14 protein	*Alternaria alternata*	1
h5	ANG83691.1	histone H3, partial	*Alternaria tenuissima*	3
h6	XP_028500422.1	ATP-dependent RNA helicase	*Alternaria arborescens*	1
h7	OWY47682.1	tuberous sclerosis 1 protein	*Alternaria alternata*	1
h8	XP_038789159.1	eukaryotic translation initiation factor 2 subunit alpha	*Alternaria burnsii*	1
h9	XP_046022265.1	high osmolarity signaling protein sho1	*Alternaria rosae*	1
h10	XP_018380507.1	bli-3 protein	*Alternaria alternata*	1
h11	OWY43746.1	ATP synthase F1 gamma	*Alternaria alternata*	1
h12				3

## Data Availability

The data presented in this study are available on request from the authors. The data are not publicly available due to privacy.
